# Reorganization of Verbal and Nonverbal Memory in Temporal Lobe Epilepsy Due to Unilateral Hippocampal Sclerosis

**DOI:** 10.1111/j.1528-1167.2007.01053.x

**Published:** 2007-08

**Authors:** H W Robert Powell, Mark P Richardson, Mark R Symms, Philip A Boulby, Pam J Thompson, John S Duncan, Matthias J Koepp

**Affiliations:** * Department of Clinical and Experimental Epilepsy, Institute of Neurology, University College LondonQueen Square, London; and MRI Unit, National Society for EpilepsyChalfont St. Peter, Bucks; † Department of Neurology, Institute of Psychiatry, Kings College LondonLondon, United Kingdom

**Keywords:** fMRI, Hippocampus, Memory, Reorganization, Temporal lobe epilepsy

## Abstract

*Purpose:* Patients with temporal lobe epilepsy (TLE) due to hippocampal sclerosis (HS) often suffer from material-specific memory impairments. The purpose of this study was to use functional magnetic resonance imaging (fMRI) to study the organization of specific memory functions in these patients.

*Methods***:** We report 14 patients with unilateral TLE and HS, and 10 controls, performing an fMRI memory paradigm of word, picture, and face encoding.

*Results:* Compared with controls, patients with left TLE demonstrated less left MTL and greater right MTL activation and patients with right TLE demonstrated less right MTL and greater left MTL activation. Correlations between fMRI activation and memory performance revealed greater activation in the damaged left hippocampus to be correlated with better verbal memory performance in left TLE patients and greater right hippocampal activation to be correlated with better nonverbal memory in right TLE patients. Conversely, greater fMRI activation in the contralateral hippocampus correlated with worse memory performance.

*Conclusions:* Our findings suggest that memory function in unilateral TLE is better when it is sustained by activation within the damaged hippocampus and that reorganization to the undamaged MTL is an inefficient process, incapable of preserving memory function.

The hippocampus and related medial temporal lobe (MTL) structures are critical for memory encoding (Squire and Zola-Morgan, 1991). Lesion deficit studies in humans have provided evidence for a material-specific lateralization of function, with the dominant (usually the left) MTL mediating verbal memory (Frisk and Milner, 1990) and nondominant (usually the right) MTL mediating nonverbal or visual memory (Smith and Milner, 1981). Functional neuroimaging combined with neuropsychological assessment allows identification of the MTL structures sustaining specific memory function (Kelley et al., 1998; Golby et al., 2001; Powell et al., 2005).

Patients with temporal lobe epilepsy (TLE) due to unilateral hippocampal sclerosis (HS) typically have medically refractory seizures but a good outcome following anterior temporal lobe resection (ATLR). Patients undergoing unilateral ATLR for refractory TLE typically show a decline in verbal memory following surgery involving the language-dominant hemisphere (Ivnik et al., 1987) and deficits in topographical memory following nondominant temporal lobe resection (Spiers et al., 2001). Prediction of memory change may reflect both the functional integrity of the to-be-resected temporal lobe as well as the capacity of the contralateral temporal lobe to maintain memory function. The known risk factors suggest that it is the functional adequacy of the resected MTL rather than the functional reserve of the contralateral MTL that determines the extent of postoperative memory decline. It therefore appears that patients with residual memory function in the pathological hippocampus are at greater risk of memory impairment postoperatively.

Functional imaging studies have demonstrated how reorganization of function occurs in response to brain injuries but recruitment of contralateral structures does not necessarily effectively maintain performance (Ward et al., 2003). While interhemispheric reorganization of language function has been shown to be associated with recovery from poststroke aphasia (Weiller et al., 1995; Blasi et al., 2002), other studies have shown recovery related to activation within unaffected ipsilateral brain regions (Heiss et al., 1999) and reorganization to the opposite hemisphere to be associated with poor recovery (Johansen-Berg et al., 2002; Ward et al., 2003). Reorganization of function also occurs in TLE due to unilateral HS with a higher incidence of atypical language dominance being demonstrated in patients with left TLE (Springer et al., 1999; Adcock et al., 2003), and earlier age of onset of epilepsy being associated with more bilateral language activation (Brazdil et al., 2005). So far, reorganization of function to the contralateral hippocampus has only been reported in patients with left HS, showing reorganization of function to the right hippocampus and parahippocampal gyrus in patients compared to controls (Richardson et al., 2003).

We describe here an fMRI study of verbal and nonverbal memory encoding in TLE patients with left or right HS, compared to normal controls, to provide evidence for reorganization of function due to HS. We hypothesized that pathology in the left MTL would result in reorganization of verbal encoding to the right MTL and that pathology in the right MTL would lead to reorganization of nonverbal encoding to the left MTL. We also looked for brain regions where functional activation correlated with performance on standard neuropsychological measures to determine whether ipsi- or contralateral structures are responsible for retaining memory function in the presence of unilateral MTL pathology.

## METHODS

### Subjects

We studied 14 patients (median age 33.5; range 25–47 year; seven female) with medically refractory TLE undergoing presurgical evaluation at the National Hospital for Neurology and Neurosurgery, London, U.K. All patients had undergone structural MRI at 1.5T (Duncan, 1997) showing left HS in seven patients and right HS in seven. Video-EEG had confirmed seizures arising from the ipsilateral medial temporal lobe in all 14. All patients had a normal contralateral hippocampus on structural imaging. Hippocampal volumetry was carried out according to a previously published protocol (Van Paesschen et al., 1995). All patients were on antiepileptic medication and all were fluent English speakers. Handedness was determined using a standardized questionnaire (Oldfield, 1971) and language dominance was assessed using a range of fMRI tasks (Powell et al., 2006). All patients had undergone standardized presurgical neuropsychological assessment (Coughlan and Hollows, 1985). Patient demographics, neurological and neuropsychological test results, and surgical outcome data are detailed in Table 1.

**TABLE 1:** Patient clinical and demographic data

Age/gender	Handedness	Age of epilepsy onset (year)	Seizure types and frequency (per month)	Postop outcome (Engel class.)	MRI and pathological diagnosis	Epilepsy syndrome	VIQ	PIQ	Verbal recall	Figure recall	Right HV (cm^3^)	Left HV (cm^3^)	Total intracranial volume (cm^3^)	Hippocampal volume ratio (%)	Language dominance
25/F	Right	17	CPS 8 SGTC 0.5	IA	Left HS	Left TLE	82	80	46	60	2.729	1.351	168.53	50	Left
37/M	Left	1	SPS 12 CPS 4	IB	Left HS	Left TLE	94	93	23	49	3.3	1.88	175	57	Left
33/M	Right	1	SPS 4 CPS 4	IA	Left HS	Left TLE	86	88	46	91	2.83	2.02	NM	71	Left
32/F	Right	22	SPS 30 CPS 5 SGTC 0.25	NA	Left HS	Left TLE	88	111	27	40	2.359	1.789	152.9	76	Left
37/F	Right	18	CPS 10	IA	Left HS	Left TLE	105	110	50	66	2.912	1.602	143.5	55	Left
28/M	Right	3	CPS 1	IA	Left HS	Left TLE	76	81	18	10	3.077	1.417	171.24	46	Left
34/M	Right	21	CPS 5	NA	Left HS	Left TLE	67	76	3	8	2.235	1.896	172.2	85	Left
25/M	Right	5	CPS 3	NA	Right HS	Right TLE	101	90	50	59	1.84	2.35	NM	78	Left
47/M	Right	13	CPS 1	IB	Right HS	Right TLE	73	86	36	18	1.038	2.769	145.92	37	Left
44/F	Right	14	CPS 4	IA	Right HS	Right TLE	95	104	64	80	2.606	3.034	154.9	86	Left
46/F	Right	8	CPS 5	IA	Right HS	Right TLE	88	84	60	13	1.756	2.695	147	65	Left
32/F	Right	19	CPS 2	IIIA	Right HS	Right TLE	87	110	80	66	2.299	2.562	139.55	90	Left
29/M	Right	9	CPS 30 SGTC 4	NA	Right HS	Right TLE	93	114	88	84	2.273	2.978	162.86	76	Left
41/F	Right	14	CPS 4	IA	Right HS	Right TLE	90	88	75	47	1.628	2.302	150.8	71	Left

M, male; F, female; CPS, complex partial seizure; SGTC, secondary generalized tonic–clonic seizure; NA, not applicable; HS, hippocampal sclerosis; EEG, electroencephalogram; TLE, temporal lobe epilepsy; VIQ, verbal intelligence quotient; PIQ, performance intelligence quotient; HV, hippocampal volume; AED, antiepileptic drug; TPR, topiramate; LTG, lamotrigine; VPA, sodium valproate; CBZ, carbamazepine; LVT, levetiracetam; PMD, primidone; CLB, clobazam; OXC, oxcarbazepine; GBP, gabapentin; PHT, phenytoin; TGB, tiagabine.

We also studied 10 right-handed native English speaking healthy volunteers (median age 30; range 23–37; five female) with no history of neurological or psychiatric disease. Results from the control subjects have been reported previously (Powell et al., 2005). The study was approved by the National Hospital for Neurology and Neurosurgery and the Institute of Neurology Joint Research Ethics Committee and informed written consent was obtained from all subjects.

### MR data acquisition

MRI studies were performed on a 1.5 T General Electric Signa Horizon scanner (Milwaukee, Wisconsin, U.S.A.). Standard imaging gradients with a maximum strength of 22 mT m ^−1^ and slew rate 120 T m^−1^ s^−1^ were used. All data were acquired using a standard quadrature birdcage head coil for both RF transmission and reception. For each subject we acquired a whole brain high resolution EPI image comprising 60 contiguous 2.3 mm slices with a 22 cm field of view, 256 × 256 matrix and in plane resolution of 0.9 × 0.9mm. The acquisition of this series was such that the geometric distortions due to susceptibility artifacts were equal to those in the images acquired in the fMRI acquisition (Boulby et al., 2004). These were spatially normalized and a mean image was calculated to display results.

For the memory task, gradient-echo echo-planar T_2_*-weighted images were acquired, providing blood oxygenation level dependent (BOLD) contrast. Each volume comprised 12 contiguous 2.3 mm oblique axial slices through the temporal lobes, with a 22 cm field of view, 96 × 96 matrix and in-plane resolution of 1.72 × 1.72 mm. TE was 40 ms and TR 4.5 s. The field of view was positioned to cover the temporal lobes with the antero-posterior axis aligned with the long axis of the hippocampus on sagittal views with the body of the hippocampus in the center. The imaging time series was realigned, normalized into standard anatomical space using the high resolution whole brain EPI, and smoothed with a Gaussian kernel of 10 mm full width half maximum.

### Psychological task

Stimuli of three different material types (pictures (P), words (W), and faces (F)) were visually presented to the subjects during a single scanning session. The pictures were black and white nameable line drawn objects (Snodgrass and Vanderwart, 1980), the words were single concrete nouns and the faces were black and white photographs unfamiliar to the subjects. A total of 210 stimuli were presented, one every 4 s, in seven cycles. Each cycle consisted of a block of 10 pictures, a block of 10 words and a block of 10 faces (each lasting 40 s) followed by 20 s of crosshair fixation. Subjects performed a deep encoding task which involved making a judgement on whether each stimulus was pleasant or unpleasant (Craik and Lockhart, 1972). Sixty minutes after scanning, subjects performed a recognition test which was not scanned; this comprised three blocks, one for each of the three material types. During each recognition block the 70 stimuli of each type presented during scanning were randomly mixed with 35 foils and presented in a manner identical to that used during scanning. Subjects were instructed to indicate whether they could remember seeing each stimulus during scanning (R response) or whether it was new (N response).

The 210 encoding stimuli that had been presented during scanning were then classified according to the responses made during the recognition test. A correct (R) response indicated the stimulus was subsequently remembered while an incorrect (N) response indicated the stimulus was subsequently forgotten. For each of the three stimulus types (P, W, and F), R and N responses were identified, giving a total of six event types: PR, PN, WR, WN, FR, and FN. These were then entered as regressors in the design matrix.

To calculate recognition accuracy, stimuli seen in the recognition test were classified as “hits” (stimuli correctly remembered) and “false alarms” (foils incorrectly tagged as remembered). Recognition accuracy was calculated for each stimulus type as: hit rate – false alarm rate.

### Data analysis

- Event-related design
  Imaging data were analyzed using Statistical Parametric Mapping (SPM2) (Friston et al., 1995). To test for subsequent memory effects, an event-related design was used to compare encoding-related responses to individual stimuli that were subsequently remembered versus stimuli that were forgotten (Friston et al., 1998). A two-level random-effect analysis was employed.
  At the first level, trial-specific responses were modeled for each subject by convolving a delta function that indicated each event onset with the canonical haemodynamic response function (HRF) to create regressors of interest, one regressor for each of the six event types described above. Each subject's movement parameters were included as confounds. Parameter estimates pertaining to the height of the HRF for each regressor of interest were calculated for each voxel. Contrasts of parameter estimates were calculated in a voxel-wise manner to produce, for each subject, one contrast image corresponding to the subsequent memory effect for each material type (PR-minus-PN, WR-minus-WN, FR-minus-FN). All these images were used for the second-level analysis.
  At the second level of the random effects analysis, we split the subjects into two groups; left TLE and right TLE. Within each group, each subject's contrast image was entered into a one-sample t-test to examine effects across that group. This was performed for the subsequent memory effect of each material type. In addition, we performed two-sample *t*-tests to compare each patient group in turn with controls. This allowed us to identify brain regions demonstrating greater or less activation in patients compared with controls.
  We report all MTL activations at a threshold of p < 0.005, uncorrected for multiple comparisons. This uncorrected threshold was adopted because of the low signal-to-noise ratio in the anterior temporal lobe (Ojemann et al., 1997; Strange et al., 2002) (Powell et al., 2005) and as we were testing a specific hypothesis regarding MTL activation. MTL regions of activation were labeled with reference to Duvernoy's *The Human Hippocampus* (Duvernoy, 1998).
- Blocked design
  In addition we performed a blocked analysis of the data. Regressors of interest were formed by creating four boxcar functions (one for pictures, one for words, one for faces, and one for crosshair fixation) convolved with the canonical HRF. Each subject's movement parameters were included as confounds and parameter estimates for the regressors were calculated for each voxel. Contrast images for each material type against fixation were created to produce, for each subject, one contrast image corresponding to the main effect of viewing each material type. Performance in the recognition test was not taken into account in this model, which therefore only assumes encoding without testing for subsequent memory effect. In the results section we refer to data from the event-related design as effects of stimulus *encoding* and from the blocked design as effects of *viewing* stimuli. All these images were used for the second-level analysis.
  At the second level of the random effects analysis, a one-sample *t*-test was performed to examine the effects of each material type across both left and right TLE groups. Two-sample *t*-tests were also performed to compare activation between patient groups and controls. We report all MTL activations at a threshold of p < 0.005, uncorrected for multiple comparisons.
- Structure–function correlations
  In order to test for correlations between areas of fMRI activation and severity of pathology, regression analyses were performed between encoding-related activation and hippocampal volume. We used left hippocampal volume as the covariate for the left HS group and right hippocampal volume as the covariate for the right HS group. To account for the variability in hippocampal volumes due to the different brain sizes of the subjects we used total intracranial volume as a second covariate of no interest in our regression analyses (Richardson et al., 2004).
- fMRI–standard memory test correlations
  We also tested for a relationship between patients' fMRI activation and their performance on routine preoperative memory assessment. For patients with left HS we used verbal recall score as a measure of verbal memory and for patients with right HS we used figure recall score as a measure of nonverbal memory (Baxendale et al., 1998). These measures of memory are routinely used in our surgical program. In the verbal recall task the subject is read a short story and recall is tested immediately and at a 30 min delay. The percentage of the story retained at 30 min was used as an indicator of verbal memory competence. In the figure recall task the subject firstly copies a complex design and reproduces it from memory immediately and at a 30 min delay. The percentage of the figure remembered following the delay was employed as the measure of nonverbal memory competence.
  In order to test for correlations with fMRI activation we defined spherical regions of interest (ROIs) (10 mm radius) in the anterior hippocampus. On the left this was centered on the coordinates of peak word encoding-related activation previously reported in controls (Powell et al., 2005). A homotopic ROI was defined on the right. We tested for correlations between patients' fMRI activation within these ROIs and their performance on the memory tests.
- Correlations with age of onset of epilepsy
  Finally we tested for correlations between age of onset of epilepsy and memory performance and between age of onset of epilepsy and fMRI activation.

## RESULTS

### Hippocampal volumes

Left and right hippocampal volumes were significantly different in both left and right TLE patients; left TLE group mean (SD) right hippocampal volume 2.78 (0.38) cm^3^, mean left hippocampal volume 1.71 (0.26) cm^3^ (paired *t*-test p = 0.001, 2-tailed); right TLE group mean right hippocampal volume 1.98 (0.63) cm^3^, mean left hippocampal volume 2.61 (0.24) cm^3^, (paired *t*-test p = 0.04, 2-tailed). There was a significant interaction between group and hippocampal volume (F_[1,12]_= 29.8, p < 0.001).

### Behavioral results

Recognition accuracy for the stimuli seen during scanning was calculated for each stimulus type in all three groups and results were compared with a 3 × 3 within-subjects ANOVA. This revealed a significant main effect of stimulus (F_[2,21]_= 106.5, p < 0.001), with better recognition accuracy for words (controls 0.64, left TLE 0.37, right TLE 0.55) and pictures (controls 0.69, left TLE 0.60, right TLE 0.61) compared to faces (controls 0.06, left TLE 0.10, right TLE 0.05). There were no significant differences between groups (F_[2,21]_= 1.9, p = 0.17). The group by stimulus interaction across all 3 groups and stimulus types was not significant (F_[4,42]_= 1.9, p = 0.12); however looking only at the performance for words and faces revealed a significant interaction between left and right TLE groups and stimulus type characterized by lower recognition accuracy for words in left TLE patients and lower recognition accuracy for faces in right TLE patients (F_[1,12]_= 20.95, p = 0.004). A subsequent memory effect was shown with recognition accuracy being significantly different from zero for all three material types (words t = 11.429, p < 0.0001, pictures t = 16.441, p < 0.0001 and faces t = 4.340, p < 0.0001).

### Imaging results

#### Controls

The results from the control subjects have been reported previously (Powell et al., 2005). Briefly, using an event-related analysis, word encoding (WR-minus-WN) was associated with activation in the left hippocampus (p = 0.002). For picture encoding (PR-minus-PN) there were bilateral areas of MTL activation in the left (p = 0.001) and right parahippocampal regions (p < 0.001), both in the depths of the collateral sulcus. Two MTL areas were associated with face encoding (FR-minus-FN); right hippocampus (p = 0.001) and right amygdala (p = 0.002). Viewing words and pictures compared with crosshair fixation, using a blocked analysis (irrespective of performance on subsequent memory test) were both associated with areas of activation in the left parahippocampal gyrus (p < 0.001), posterior to the regions implicated in the event-related design. Viewing faces was associated with activation in bilateral fusiform gyri (left p < 0.001, right p = 0.002) but no hippocampal activation.

#### Left TLE

**FIG. 1:**
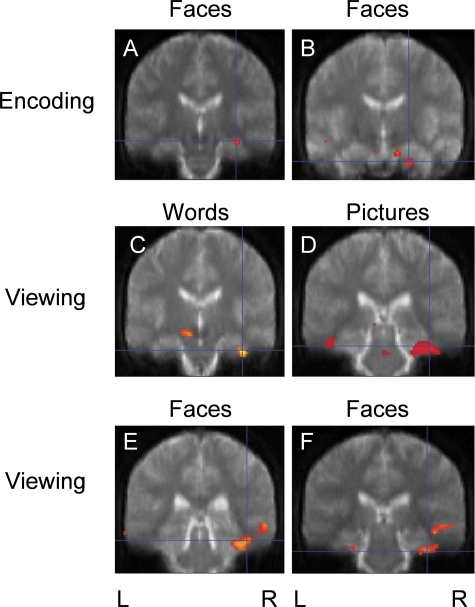
Left TLE group: event-related (encoding) and blocked (viewing) designs. Significant regions are superimposed onto the normalized mean EPI image from 10 healthy control subjects. Left (L) and right (R) side of the brain are indicated. (**A**) Main effect of face encoding, right hippocampal activity. (**B**) Main effect of face encoding, right entorhinal cortex activity. (**C**) Main effect of viewing words, right parahippocampal gyrus activity. (**D**) Main effect of viewing pictures, right parahippocampal gyrus activity. (**E**) Main effect of viewing faces, right fusiform gyrus activity. (**F**) Main effect of viewing faces, right parahippocampal gyrus activity. No significant MTL activation was found for word or picture encoding.

We examined the main effects of subsequent memory for each stimulus type (Table 2). No significant MTL activation was seen for word or picture encoding. Face encoding was associated with areas of activation in the right hippocampus (p = 0.001, Fig. 1A) and entorhinal cortex (p = 0.001, Fig. 1B).

**TABLE 2:** *Activation peaks in the medial temporal lobe for left and right TLE groups. For each effect, the Montreal Neurological Institute (MNI) coordinate,* z-*score, anatomical location and relevant figure are given*

Group	Material	Contrast	Figure	MNI coordinates	*z*-score	Region
Left TLE	Faces	Remembered > forgotten	Figure 1A	34–24–16	2.99	Right hippocampus
	Faces	Remembered > forgotten	Figure 1B	22–14–32	2.98	Right entorhinal cortex
	Words	Viewing > fixate	Figure 1C	40–20–30	4.07	Right parahippocampal gyrus
	Pictures	Viewing > fixate	Figure 1D	40–32–24	3.72	Right parahippocampal gyrus
	Faces	Viewing > fixate	Figure 1E	38–36–24	3.33	Right fusiform gyrus
	Faces	Viewing > fixate	Figure 1F	38–30–30	3.10	Right parahippocampal gyrus
Right TLE	Words	Remembered > forgotten	Figure 2A	−30–10–36	3.33	Left hippocampus
	Pictures	Remembered > forgotten	Figure 2B	−34–34–18	2.6	Left parahippocampal gyrus
	Pictures	Viewing > fixate	Figure 2C	−40–20–26	4.19	Left hippocampus
	Faces	Viewing > fixate	Figure 2D	16–18–26	4.07	Right hippocampus

The blocked analysis demonstrated that viewing words and pictures was associated with activation in the right parahippocampal gyrus (p < 0.001, Figs. 1C and D). Viewing faces was associated with activation in the right fusiform and parahippocampal gyri (p < 0.001, Figs. 1E and F).

#### Right TLE

**FIG. 2:**
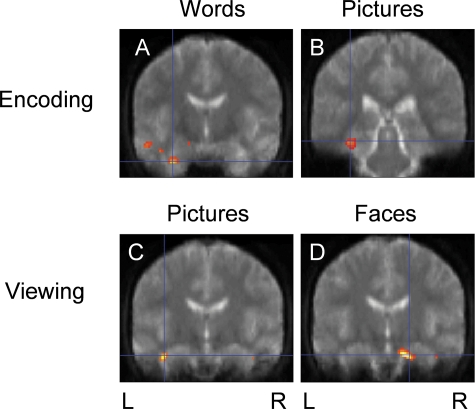
Right TLE group: event-related (encoding) and blocked (viewing) designs. (**A**) Main effect of word encoding, left hippocampal activity. (**B**) Main effect of picture encoding, left parahippocampal gyrus activity. (**C**) Main effect of viewing pictures, left hippocampal activity. (**D**) Main effect of viewing faces, right hippocampal activity. No significant MTL activation was seen for face encoding or viewing words.

Word encoding was associated with activation in the left hippocampus (p <0 .001, Fig. 2A) and picture encoding was associated with activation in the left parahippocampal gyrus (p = 0.005, Fig. 2B). No significant MTL activation was seen for face encoding.

There was no significant MTL activation for viewing words. Viewing pictures was associated with activation in the left hippocampus (p < 0.001, Fig. 2C) and viewing faces was associated with activation in the right hippocampus (p < 0.001, Fig. 2D).

### Group comparisons with controls

We performed two-sample *t*-tests to highlight brain regions demonstrating more or less activation in patient groups compared to normal controls. This was done for the results of both the event-related and blocked analysis (Table 3).

**TABLE 3:** *Activation peaks in the medial temporal lobe for group comparisonsFor each effect, the Montreal Neurological Institute (MNI) coordinate*, z*-score, anatomical location and relevant figure are given.*

Group comparison	Material	Contrast	Figure	MNI coordinates	*z*-score	Region
Left TLE > Controls	Words	Remembered > forgotten	Figure 3A	34–26–28	2.60	Right parahippocampal gyrus
Left TLE > Controls	Faces	Remembered > forgotten	Figure 3B	26–22–30	3.15	Right entorhinal cortex
Left TLE < Controls	Words	Viewing > fixate	Figure 3C	−28–28–16	3.78	Left hippocampus
Left TLE > Controls	Words	Viewing > fixate	Figure 3D	38–20–30	3.04	Right parahippocampal gyrus
Left TLE < Controls	Pictures	Viewing > fixate	Figure 3E	−26–30–18	4.44	Left hippocampus
Left TLE > Controls	Faces	Viewing > fixate	Figure 3F	44–36–18	3.40	Right fusiform gyrus
Right TLE > Controls	Words	Remembered > forgotten	Figure 4A	−18–14–28	3.07	Left hippocampus
Right TLE > Controls	Words	Remembered > forgotten	Figure 4B	−28–6–30	3.28	Left amygdala
Right TLE > Controls	Words	Viewing > fixate	Figure 4C	−40–18–26	2.99	Left hippocampus
Right TLE > Controls	Pictures	Viewing > fixate		−40–16–30	3.60	Left hippocampus

#### Left TLE

**FIG. 3:**
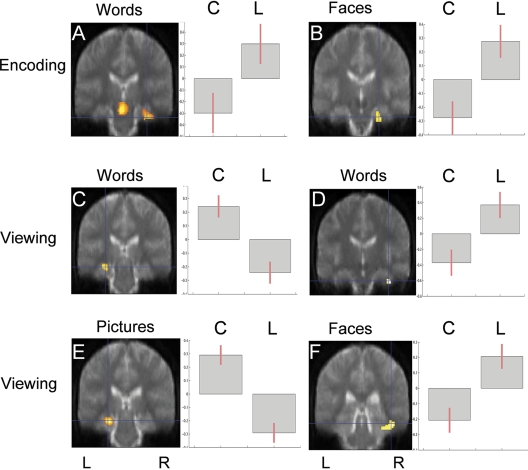
Group comparisons highlighting regions showing significant differences between left TLE patients and controls. Contrast estimates are shown on the right of the SPMs. For all graphs, controls (C) are on the left and patients (L) on the right. (**A**) Word encoding; greater activity in left TLE than in controls in the right parahippocampal gyrus. (**B**) Face encoding: greater activity in left TLE than in controls in the right entorhinal cortex. (**C**) Viewing words: less activity in left TLE than in controls, left hippocampus. (**D**) Viewing words: greater activity in left TLE than in controls, right parahippocampal gyrus. (**E**) Viewing pictures: less activity in left TLE than in controls, left hippocampus. (**F**) Viewing faces: greater activity in left TLE than in controls, right fusiform gyrus.

For both word encoding (p < 0.005, Fig. 3A) and face encoding (p = 0.001, Fig. 3B) left TLE patients showed greater activation in the right parahippocampal gyrus than controls. No significant differences were seen in the MTL between controls and the left TLE group for picture encoding.

For viewing words, left TLE patients showed less activation in the left hippocampus (p < 0.001, Fig. 3C) and greater activation in the right parahippocampal gyrus (p = 0.001, Fig. 3D) compared with controls. Left TLE patients showed less activation in the left hippocampus for viewing pictures (p < 0.001, Fig. 3E), and greater activation in the right fusiform gyrus for viewing faces (p < 0.001, Fig. 3F), compared with controls.

#### Right TLE

**FIG. 4:**
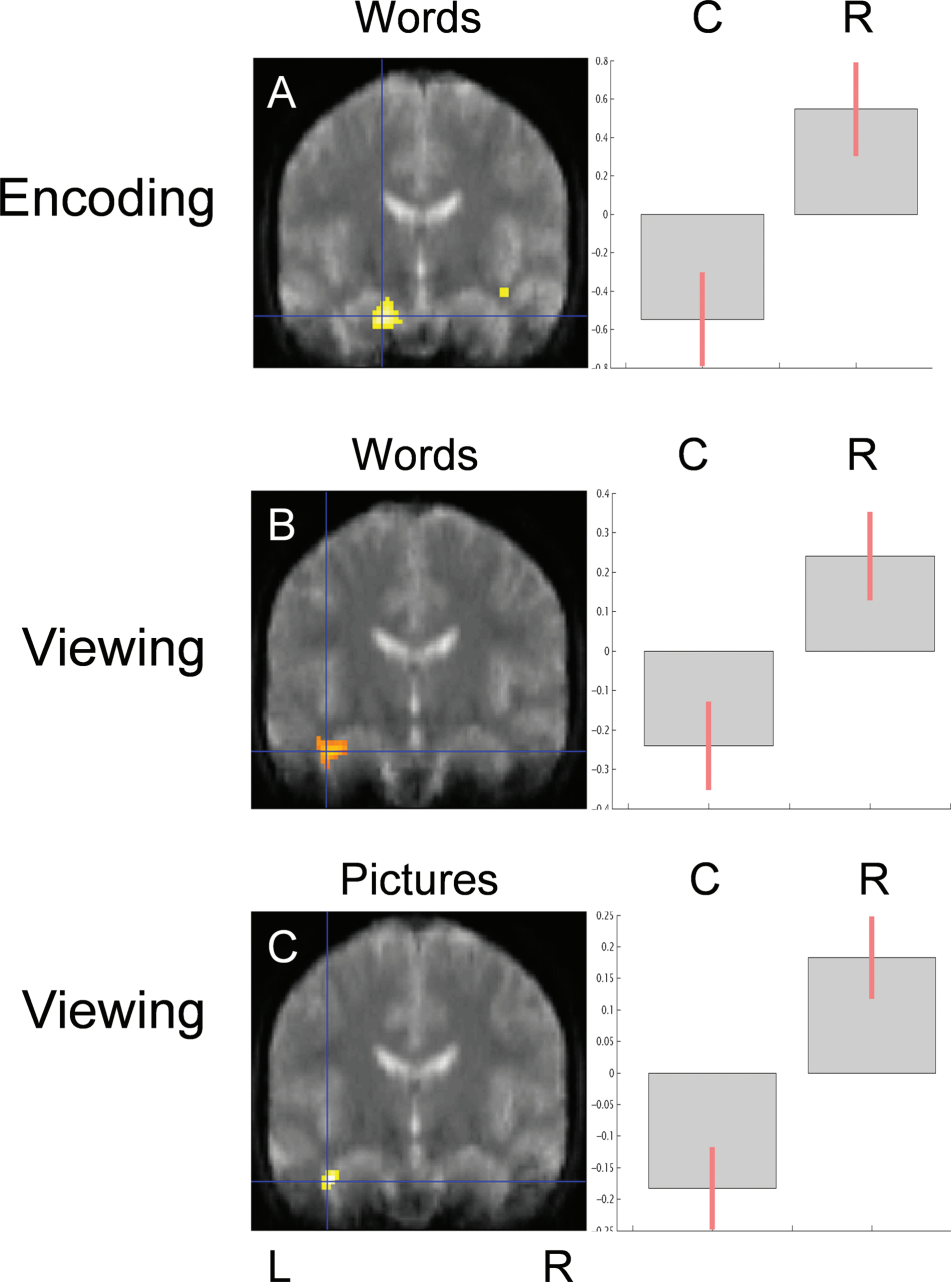
Group comparisons highlighting regions showing significant differences between right TLE patients and controls. Contrast estimates are shown on the right of the SPMs. For all graphs, controls (C) are on the left and patients (R) on the right. (**A**) Word encoding: greater activity in right TLE than in controls, left hippocampus. (**B**) Viewing words: greater activity in right TLE than in controls, left hippocampus. (**C**) Viewing pictures: greater activity in right TLE than in controls, left hippocampus.

For word encoding right TLE patients showed greater activation than controls in both the left hippocampus (p = 0.001, Fig. 4A) and left amygdala (p = 0.001). No significant differences were seen in the MTL between right TLE patients and controls for picture or face encoding. Right TLE patients showed greater activation in the left hippocampus for viewing words (p = 0.001, Fig. 4B) and pictures (p < 0.001, Fig. 4C), compared with controls.

### Structure–function correlations

**FIG. 5:**
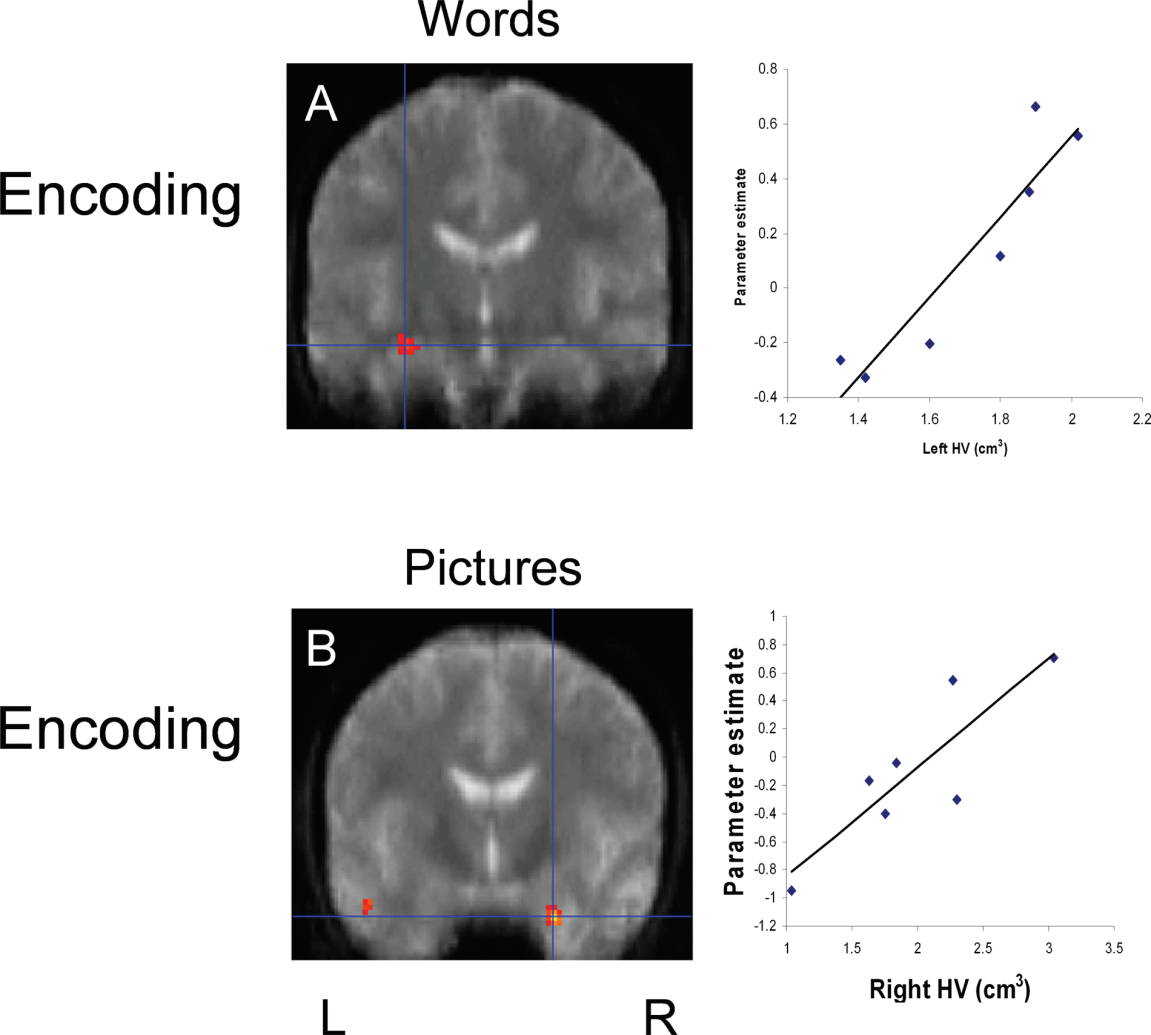
Structure–function relationships. Graphs on the right of the SPMs demonstrate the correlation between hippocampal volume (cm^3^) and fMRI activation (arbitrary units). (**A**) Positive correlation in the left hippocampus between left hippocampal volume and word encoding-related fMRI activation. (**B**) Positive correlation in the right hippocampus between right hippocampal volume and picture encoding-related fMRI activation.

We found a positive correlation between left hippocampal volume and encoding-related activation for words with less activation in the left hippocampus if left hippocampal atrophy was more severe (r = 0.885; p = 0.001, Fig. 5A, Table 4). A positive correlation was also seen between right hippocampal volume and encoding-related fMRI activation for pictures with more severe pathology leading to reduced activation in the right hippocampus (r = 0.865; p = 0.001, Fig. 5B, Table 4). The inverse correlations were not seen in the contralateral hippocampi and no correlations were observed between face encoding and hippocampal volume.

**TABLE 4:** *Activation peaks in the medial temporal lobe for structure–function correlations.For each effect, the Montreal Neurological Institute (MNI) coordinate,* z*-score, anatomical location and relevant figure are given*

Group	Correlation	Figure	MNI coordinates	*z*-score	Region
Structure–function correlations	Word encoding vs. left HV	Figure 5A	−30–18–18	3.04	Left hippocampus
	Picture encoding vs. right HV	Figure 5B	24–8–32	3.18	Right hippocampus

### Preoperative standard memory test correlations

Regression analyses were performed to examine the relationship between left and right hippocampal fMRI activation and performance on preoperative memory tests.

#### Left TLE

**FIG. 6:**
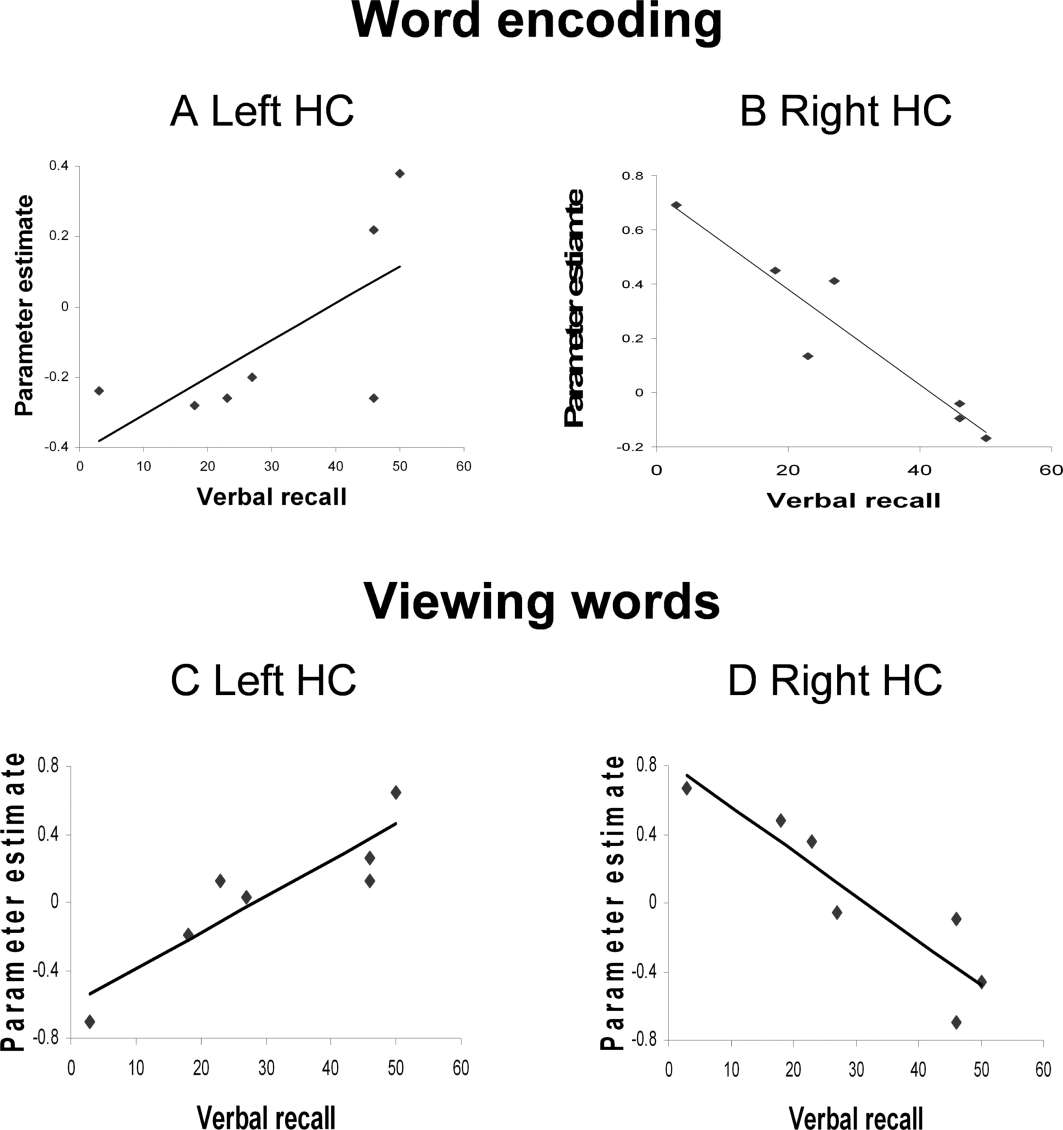
Relationships between fMRI activation and standard memory test results for the left TLE group. The graphs demonstrate the correlation between test performance and fMRI activation (arbitrary units). HC: hippocampus. (**A**) Positive correlation in the left hippocampus between verbal recall score and word encoding-related fMRI activation. (**B**) Negative correlation in the right hippocampus between verbal recall score and word encoding-related fMRI activation. (**C**) Positive correlation in the left hippocampus between verbal recall score and fMRI activation for viewing words. (**D**) Negative correlation in the right hippocampus between verbal recall score and fMRI activation for viewing words.

For word encoding there was a trend for increased left hippocampal activation to correlate with better verbal recall but this was not significant (r = 0.680; p = 0.09, Fig. 6A). A negative correlation was seen between right hippocampal fMRI activation and verbal recall (r =−0.948; p = 0.001, Fig. 6B).

For viewing words there was a positive correlation between left hippocampal fMRI activation and verbal recall performance (r = 0.898; p = 0.006, Fig. 6C) and a negative correlation was demonstrated between right hippocampal activation and verbal recall (r =−0.914; p = 0.004, Fig. 6D).

#### Right TLE

**FIG. 7:**
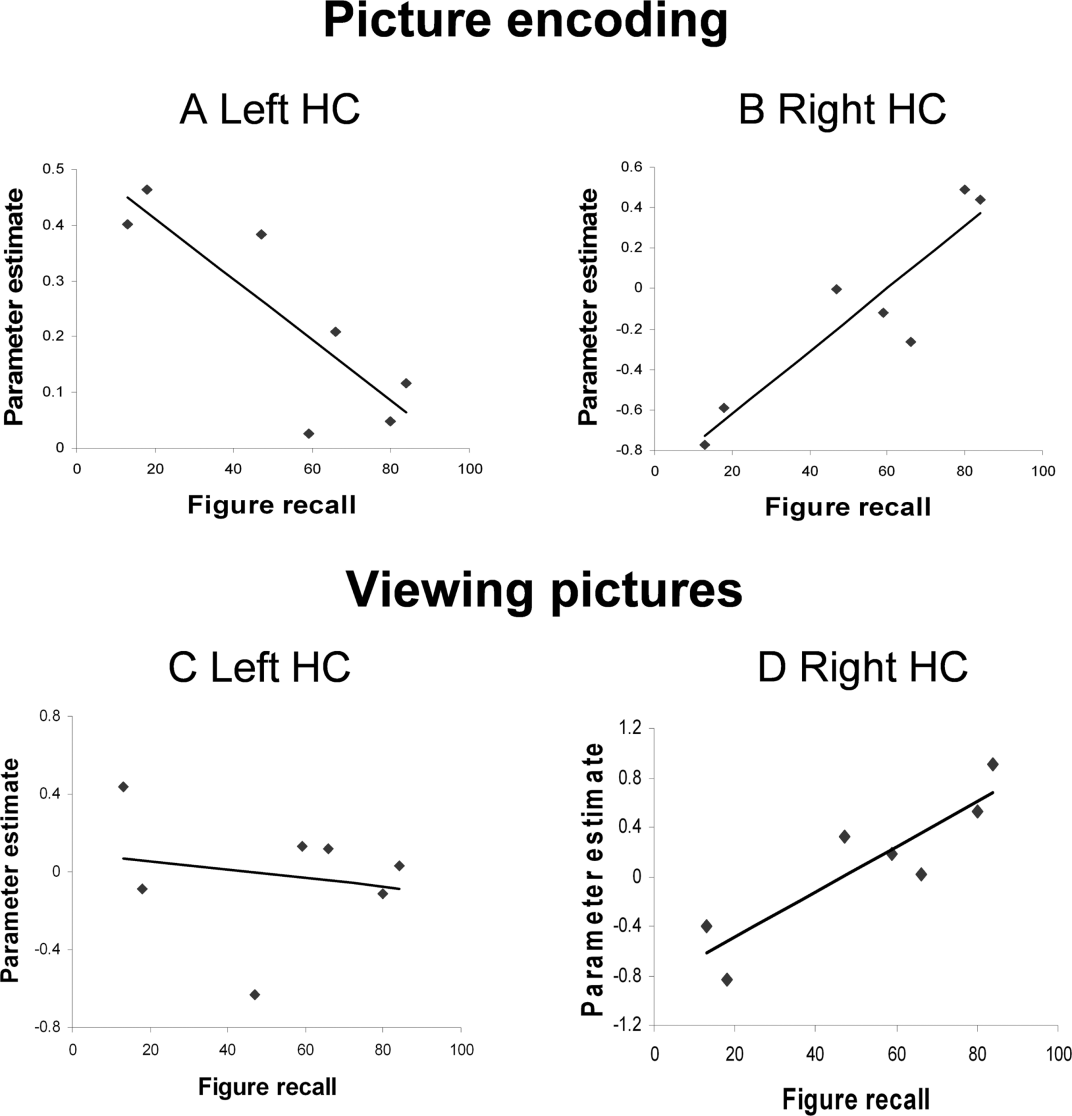
Relationships between fMRI activation and standard memory test results for the right TLE group. HC: hippocampus. (**A**) Negative correlation in the left hippocampus between figure recall score and picture encoding-related fMRI activation. (**B**) Positive correlation in the right hippocampus between figure recall score and picture encoding-related fMRI activation. (**C**) No significant correlation in the left hippocampus between figure recall score and fMRI activation for viewing pictures. (**D**) Positive correlation in the right hippocampus between figure recall score and fMRI activation for viewing pictures.

For picture encoding, there was a negative correlation between left hippocampal fMRI activation and figure recall performance (r =−0.849; p = 0.016, Fig. 7A) and a positive correlation was demonstrated between right hippocampal activation and figure recall (r = 0.916; p = 0.004, Fig. 7B). No significant correlations were seen for face encoding. For viewing pictures, there was no correlation between left hippocampal fMRI activation and figure recall (r =−0.190; p = 0.684, Fig. 7C) but a positive correlation between right hippocampal fMRI activation and figure recall performance (r = 0.891; p = 0.007, Fig. 7D). No significant correlations were seen for viewing faces.

### Correlations with age of onset of epilepsy

No correlations were seen between age of onset of epilepsy and either memory performance or fMRI activation.

## DISCUSSION

### Summary of results

In patients with unilateral TLE due to HS we found a reorganization of material-specific memory encoding (Table 5). In contrast to the left sided MTL activation associated with word and picture encoding in normal control subjects, in patients with left TLE the main effect of viewing words and pictures was seen in the right MTL. For face encoding and viewing faces MTL activation remained right-sided. When compared directly with controls, left TLE patients had less left MTL activation for viewing words and pictures and greater right MTL activation for both word and face encoding. In patients with right TLE the right-sided MTL activation seen for picture and face encoding in controls was not seen. The main effect of viewing faces remained in the right MTL. When compared directly with controls, right TLE patients demonstrated greater activity in the left MTL for word encoding, viewing words, and viewing pictures. In summary these results demonstrate less left MTL and greater right MTL activation in the left TLE patients and less right MTL and greater left MTL activation in the right TLE patients compared with controls.

**TABLE 5:** Results summary

Group	Material	Contrast	Region of main effect	Comparison with controls (increased/decreased, location)	Correlation with memory performance (location, correlating measure)
Controls	Words	Remembered > forgotten	Left HC		
	Pictures		Bilateral PHG		
	Faces		Right HC, A		
	Words	Viewing > fixate	Left PHG		
	Pictures		Left PHG		
	Faces		Bilateral FG		
Left TLE	Words	Remembered > forgotten	–	Increased, Right PHG	Right HC, 1/verbal recall
	Pictures		–	–	–
	Faces		Right HC, ERC	Increased, Right ERC	-
	Words	Viewing > fixate	Right PHG	Reduced, Left HC	Left HC, verbal recall
				Increased, Right PHG	Right HC, 1/verbal recall
	Pictures		Right PHG	Reduced, Left HC	Left HC, verbal learning
					Right HC, 1/verbal learning
	Faces		Right PHG, FG	Increased, Right FG	-
Right TLE	Words	Remembered > forgotten	Left HC	Increased, Left HC	-
	Pictures		Left PHG	-	Right HC, figure recall
	Faces		-	-	Left HC, 1/figure recall
					-
	Words	Viewing > fixate	-	Increased, Left HC	-
	Pictures		Left HC	Increased, Left HC	Right HC, figure recall
	Faces		Right HC	-	-

Summary of all the results including main effect of encoding and viewing each stimulus type for each group, comparison between patient and controls, and correlations with memory performance. HC, hippocampus; PHG, parahippocampal gyrus; A, amygdala; FG, fusiform gyrus; ERC, entorhinal cortex.

Secondly, we demonstrated that greater activation in the damaged left hippocampus is correlated with better verbal memory test performance in left TLE patients. Similarly, greater activation in the damaged right hippocampus correlated with better nonverbal memory in right TLE patients (Table 5). This suggests that material-specific memory in TLE is better when it is sustained by functional activation within the damaged hippocampus. This would be consistent with the observation that preoperative memory performance is a predictor of postoperative memory decline, with better performance predicting worse decline (Helmstaedter and Elger, 1996; Jokeit et al., 1997). The converse is true in the contralateral MTL, with greater fMRI activation correlating with worse memory performance. This suggests that although there is to some extent a compensatory increase in activation on the unaffected side this is an inefficient process and does not lead to the preservation of memory function.

Finally we demonstrated correlations between hippocampal volume and fMRI activation. This relationship between structure and function has previously been demonstrated with left hippocampal pathology predicting the magnitude of fMRI activation for word encoding, with less severe left hippocampal pathology being associated with more left hippocampal activation (Richardson et al., 2004). Our findings replicate and extend this to show a similar correlation between right hippocampal volume and right hippocampal activation for picture encoding. In summary it appears that more severe hippocampal pathology is associated with worse memory and with less ipsilateral and greater contralateral activation. The extent of the functional reorganization that takes place therefore appears to be proportional to the degree of damage to the hippocampus.

### Limitations of the study

Partial volume effects may contribute to the findings. A sclerotic hippocampus will contain fewer voxels that may demonstrate a BOLD effect. Our study focused on reorganization within MTL structures and our choice of imaging parameters and statistical thresholds were based on this preexisting hypothesis (Powell et al., 2005). It is possible that reorganization may take place to other neocortical regions both within and outside the temporal lobes; however we were unable to assess the degree of extratemporal reorganization that may have occurred in these patients. Our findings also refer to a relatively small sample of patients and the findings will require confirmation in larger groups. However our results replicate and extend previous fMRI findings in an independent patient sample (Richardson et al., 2003, 2004), and are consistent with clinical findings from patients undergoing ATLR.

### Memory fMRI in TLE

Several studies have used fMRI in the assessment of memory function in patients with unilateral TLE. A blocked design experiment comparing presentation of complex visual scenes with randomly “scrambled” pictures demonstrated symmetrical MTL activation in controls and asymmetric MTL activation concordant with results from intracarotid amytal testing (IAT) in patients with unilateral TLE (Detre et al., 1998). Greater left MTL activation was observed in patients with right TLE than left TLE using a semantic decision task, where an assumption of incidental encoding was made (Bellgowan et al., 1998). Dupont et al. demonstrated different patterns of activation in patients with left TLE compared to controls during memory encoding and retrieval of word lists (Dupont et al., 2000). An area of activation in the posterior parahippocampal gyrus was identified to be greater in controls than in patients; however there was a highly significant difference in task performance between the two groups, with patients performing the task poorly, which limits the interpretation. Mental navigation of a familiar spatial environment has been shown to result in bilateral symmetric pattern of MTL activation in controls but an asymmetric pattern of activation in patients with unilateral TLE with reduced activation ipsilateral to the affected MTL (Jokeit et al., 2001). Finally, Golby et al. used a blocked design experiment comparing novel versus repeated stimuli, finding that the side of the epileptic focus influenced the lateralization of fMRI activation within the MTL, with greater activation contralateral to the affected side (Golby et al., 2002).

Some of these studies only revealed areas of activation in posterior MTL, used blocked experimental designs which involved an assumption of memory encoding, did not perform direct comparisons with normal subjects, and (while showing regions of reduced activation) did not demonstrate any reorganization of function to other brain regions. Using an event-related design, Richardson et al. demonstrated a reorganization of verbal memory encoding from left to right MTL in left TLE patients however nonverbal memory was not assessed in this study (Richardson et al., 2003). Our study extends these findings by assessing both verbal and nonverbal memory and patients with left and right TLE and by looking at correlations between functional activation and memory performance.

### Hippocampal functional adequacy versus functional reserve

Two different models of hippocampal function have been proposed to explain memory deficits following ATLR; hippocampal reserve and functional adequacy (Chelune, 1995). According to the hippocampal reserve theory, postoperative memory decline depends on the capacity or reserve of the contralateral hippocampus to support memory following surgery, while the functional adequacy model suggests that it is the capacity of the hippocampus that is to be resected that determines whether changes in memory function will be observed. Evidence from baseline neuropsychology (Chelune et al., 1991), intracarotid amytal testing (IAT) (Kneebone et al., 1995), histological studies of hippocampal cell density (Sass et al., 1990), and MRI volumetry (Trenerry et al., 1993) has suggested that of the two, it is the functional adequacy of the ipsilateral MTL, rather than the functional reserve of the contralateral MTL that is most closely related to the typical material-specific memory deficits seen following ATLR.

By correlating performance on standard neuropsychological tests with memory-related fMRI activation we demonstrated that MTL activation ipsilateral to the pathology is correlated with better performance while contralateral, compensatory activation correlates with poorer performance. This suggests that reorganization to contralateral MTL structures is not an effective way of maintaining memory function. The conclusion that memory function in unilateral TLE is better when sustained by the activation within the damaged hippocampus adds further support to the functional adequacy model of hippocampal function.

We have reported a group of consecutive presurgical patients with TLE and HS all of whom were left hemisphere dominant for language. Patients with atypical language organization may also have atypical lateralization of material-specific memory, and while it appears to be the case that in this study memory function was sustained by activation within the damaged hippocampus, this may not apply to patients with atypical dominance.

The same principle may also be relevant to the reorganization of language function in TLE. Helmstaedter et al. studied patterns of language dominance, as assessed by IAT, in TLE patients. They found that in patients with left TLE, atypical language dominance was associated with poorer language function, again suggesting that reorganization of function is an inefficient process (Helmstaedter et al., 1997).

### Functional reorganization

Functional imaging studies have demonstrated reorganization of function in the presence of other neurological disease, including stroke (Warburton et al., 1999; Blasi et al., 2002; Johansen-Berg et al., 2002), MS (Filippi and Rocca, 2004), and peripheral motor denervation (Reddy et al., 2002). A number of these suggest that functional reorganization to the contralateral hemisphere takes place but it is not clear whether this necessarily provides an effective mechanism of maintaining performance. Contradictory evidence has emerged from studies of stroke patients with interhemispheric reorganization of language function associated with recovery from poststroke aphasia in some studies (Weiller et al., 1995; Blasi et al., 2002), while others have shown clinical recovery related to activation within unaffected ipsilateral brain regions (Heiss et al., 1999), and reorganization to the opposite hemisphere to be associated with poor recovery (Warburton et al., 1999; Johansen-Berg et al., 2002; Ward et al., 2003). It is only by correlating fMRI with behavioral measures that is it possible to demonstrate brain regions where functional activation correlates with better performance. In a study of patients with intact reading skills following left ATLR, Noppeney et al. used reading scores in patients who had had left ATLR as covariates in order to identify brain regions where activation increased with reading ability (Noppeney et al., 2005). The patients' reading skills relied upon integration of regions from the normal (bilateral) reading system along with recruitment of other right hemisphere regions. In a study of patients with Alzheimer's disease Golby et al. also found correlations between behavioral measures of memory function and activation in posterior MTL, lingual and fusiform areas (Golby et al., 2005).

Our findings suggest that reorganization to contralateral MTL structures is not an effective way of maintaining memory function and are analogous to those of Ward et al. in patients recovering motor function following stroke. Patients with more complete recovery were more likely to have “normal” motor activation whereas activation in other motor-related brain regions correlated negatively with outcome (Ward et al., 2003).

In a retrospective study of the factors influencing language reorganization in patients with TLE due to HS, who had undergone presurgical evaluation, it was found that atypical speech representation was associated with higher interictal spiking frequency and with sensory auras suggesting ictal involvement of the lateral temporal structures (Janszky et al., 2003). This suggested that functional factors such as the epileptic activity itself may influence language organization in TLE, as well as structural factors.

### Clinical implications

The ability to predict the effect of left or right ATLR on verbal and nonverbal memory in an individual patient is an important part of the presurgical evaluation. Current predictors of postoperative memory outcome include the severity of HS on preoperative structural imaging with larger hippocampal volume being correlated with a decline in verbal memory following resection (Trenerry et al., 1993). Preoperative memory performance on neuropsychological testing has been related to degree of postoperative memory impairment, with better performance increasing the risk of clinically significant memory decline (Chelune et al., 1991; Helmstaedter and Elger, 1996; Jokeit et al., 1997). Our findings demonstrate that both hippocampal volume and memory performance are correlated with functional activation within the to-be-resected hippocampus, hence explaining why these are risk factors for memory decline following ATLR.

### Summary

In summary, using a paradigm that allows the assessment of verbal and nonverbal memory in a single scanning session, we have demonstrated a partial reorganization of verbal and nonverbal memory in patients with both left and right TLE due to HS, with the extent of reorganization being proportional to the degree of hippocampal damage. We also demonstrated that good memory performance is sustained by activation within the pathological MTL, explaining why patients with better preoperative memory functioning are most at risk of postoperative memory impairment. We anticipate that this information, in combination with structural MRI to evaluate hippocampal pathology and baseline neuropsychology, will enable preoperative prediction of material-specific memory impairments seen following unilateral ATLR to be made with greater accuracy. The patients reported here are currently being followed up and we will report changes in their verbal and nonverbal memory and its relation to the fMRI results in due course.
